# Complete Genome Sequences of Two *Salmonella enterica* subsp. *enterica* Serovar Enteritidis Strains Isolated from Egg Products in the United States

**DOI:** 10.1128/genomeA.00614-17

**Published:** 2017-06-29

**Authors:** Lijun Hu, Guodong Zhang, Marc W. Allard, Kuan Yao, Robert Stones, Maria Hoffmann, Eric W. Brown

**Affiliations:** a Division of Microbiology, Office of Regulatory Science, Center for Food Safety and Nutrition, U.S. Food and Drug Administration, College Park, Maryland, USA; b Newcastle University, Newcastle upon Tyne, United Kingdom

## Abstract

Egg-associated salmonellosis is an important public health problem in many countries. Here, we report the genome sequences, including plasmids, of two strains of *Salmonella enterica* subsp. *enterica* serovar Enteritidis isolated from egg products in 2012 and 2013 in the United States. This will provide more information and insight into the research about egg-associated salmonellosis.

## GENOME ANNOUNCEMENT

*Salmonella enterica* subsp. *enterica* serovar Enteritidis is a significant threat to public health worldwide (1). The Centers for Disease Control and Prevention (CDC) reported that *S.* Enteritidis was the most frequently detected causative *Salmonella* serovar in foodborne outbreaks reported in the United States in 2015 (18% of total confirmed *Salmonella* infections), and the incidence rate was 2.83 per 100,000 persons (2). Egg and egg products were the most common identified food vehicles associated with *Salmonella* outbreaks. The CDC estimated that approximately one in 10,000 eggs may be internally contaminated in the northeast of the United States (3). In the European Union, *S.* Enteritidis remained the most commonly reported *Salmonella* serovar in confirmed human cases in 2013, representing 39.5% of all reported serovars. In addition, 44.9% of all *Salmonella* outbreaks were associated with eggs and egg products (4). The target gene (*prot*6E) of frequently used molecular detection methods for *S.* Enteritidis is located on a plasmid. A complete genome sequence, including plasmids, will be very beneficial to designing new detection methods for *S.* Enteritidis.

In this announcement, we report the complete genomes, including plasmids, of two *S.* Enteritidis strains isolated from egg products in the United States: CFSAN033543 (Ohio, raw whole eggs, 2012) and CFSAN033541 (Pennsylvania, raw egg whites, 2013). Both strains displayed the pulsed-field gel electrophoresis pattern of JEGX01.0004.

*S.* Enteritidis strains were cultured overnight at 37 ± 2°C in tryptic soy broth (Becton, Dickinson, Franklin Lakes, NJ, USA). The genomic DNA was extracted using the DNeasy blood and tissue kit (Qiagen, Valencia, CA, USA), and then sequenced using the Pacific biosciences (PacBio) RS II sequencing platform (5, 6). The PacBio hierarchical genome assembly process (HGAP version 3.0) was used for the *de novo* assembly of the sequence reads (7). Draft genomes of the two strains were annotated using the NCBI Prokaryotic Genome Annotation Pipeline (PGAP) and deposited at DDBJ/EMBL/GenBank.

The genomes of the CFSAN033541 and CFSAN033543 chromosomes have been fully closed with 380× and 332× coverages, respectively, while the CFSAN033541 and CFSAN033543 plasmid genomes have been fully closed with 130× and 120× coverages, respectively. The complete genome of the CFSAN033541 chromosome and plasmid consisted of 4,679,064 bp and 59,372 bp, with G+C contents of 52.50% and 53.01% and with 4,511 and 74 genes, respectively. The complete genome of the CFSAN033543 chromosome and plasmid consisted of 4,679,401 bp and 59,363 bp, with G+C contents of 52.51% and 53.12% and with 4,514 and 74 genes, respectively. The PHAge Search Tool (PHAST) analysis for prophage sequence detection identified the presence of prophages Escher-Sakai and Escher-EDL933 in both sequenced *S.* Enteritidis strains (8).

### Accession number(s).

The complete genome and plasmid sequences of strains CFSAN033541 and CFSAN033543 have been deposited in NCBI/GenBank. The accession numbers for the complete genomes of CFSAN033541 and CFSAN033543 are CP020823 and CP020825, respectively. The accession numbers for the plasmids are CP020824 and CP020826, respectively.
